# Trimetazidine enhances myocardial angiogenesis in pressure overload-induced cardiac hypertrophy mice through directly activating Akt and promoting the binding of HSF1 to VEGF-A promoter

**DOI:** 10.1038/s41401-022-00877-8

**Published:** 2022-02-25

**Authors:** Hong-yang Shu, Yi-zhong Peng, Wei-jian Hang, Min Zhang, Lan Shen, Dao-wen Wang, Ning Zhou

**Affiliations:** 1 Division of Cardiology, Department of Internal Medicine, Tongji Hospital, Tongji Medical College, Huazhong University of Science and Technology, Wuhan, 430030 China; 2 Hubei Key Laboratory of Genetics and Molecular Mechanism of Cardiologic Disorders, Huazhong University of Science and Technology, Wuhan, 430030 China; 3 Department of Orthopedics, Union Hospital, Tongji Medical College, Huazhong University of Science and Technology, Wuhan, 430030 China; 4 Department of Cardiology, Shanghai Chest Hospital Shanghai Jiaotong University, Shanghai, 200030 China

**Keywords:** cardiac angiogenesis, pressure overload-induced cardiac hypertrophy, HSF1, VEGF-A, Akt, trimetazidine

## Abstract

Latest clinical research shows that trimetazidine therapy during the perioperative period relieves endothelial dysfunction in patients with unstable angina induced by percutaneous coronary intervention. In this study we investigated the effects of TMZ on myocardial angiogenesis in pressure overload-induced cardiac hypertrophy mice. Cardiac hypertrophy was induced in mice by transverse aortic constriction (TAC) surgery. TAC mice were administered trimetazidine (2.8 mg/100 µL, i.g.) for 28 consecutive days. We showed that trimetazidine administration significantly increased blood vessel density in the left ventricular myocardium and abrogated cardiac dysfunction in TAC mice. Co-administration of a specific HSF1 inhibitor KRIBB11 (1.25 mg/100 µL, i.h.) abrogated the angiogenesis-promoting effects of trimetazidine in TAC mice. Using luciferase reporter and electrophoretic mobility shift assays we demonstrated that the transcription factor HSF1 bound to the promoter region of VEGF-A, and the transcriptional activity of HSF1 was enhanced upon trimetazidine treatment. In molecular docking analysis we found that trimetazidine directly bound to Akt via a hydrogen bond with Asp292 and a pi–pi bond with Trp80. In norepinephrine-treated HUVECs, we showed that trimetazidine significantly increased the phosphorylation of Akt and the synergistic nuclear translocation of Akt and HSF1, as well as the binding of Akt and HSF1 in the nucleus. These results suggest that trimetazidine enhances myocardial angiogenesis through a direct interaction with Akt and promotion of nuclear translocation of HSF1, and that trimetazidine may be used for the treatment of myocardial angiogenic disorders in hypertensive patients.

## Introduction

It is estimated that about 29.2% of the global population will suffer from hypertension by 2025. However, the control rate of hypertension is <15% [1–3]. The huge hypertensive population has produced 64.3 million heart failure patients, whose 5-year mortality rate exceeds 50% [4, 5]. Heart failure also imposes a huge economic burden, estimated at $108 billion per annum [6]. Therefore, exploring the molecular mechanisms and drugs related to hypertension and heart failure has always been an important topic in clinical and scientific communities.

The rarefaction of capillary density causes myocardial hypoxia and contractile dysfunction. Angiogenesis dysfunction caused by pressure overload is a critical pathological process that deteriorates cardiac function [7]. Capillaries and myocytes grow in proportion to the increasing heart mass in physiological cardiac hypertrophy, and the disproportionate growth of capillaries and myocytes during pressure overload contributes to the formation of pathological cardiac hypertrophy and its transition to heart failure [8]. Thus, ameliorating angiogenesis dysfunction may provide a promising therapeutic strategy for the treatment of cardiac hypertrophy and heart failure.

Trimetazidine (TMZ) has been used in Europe for more than 40 years, and has been recommended as a second-line drug for stable angina pectoris by the European Society of Cardiology [9]. The accumulated evidence suggests that TMZ is not only effective for angina pectoris, but also for heart failure [10], peripheral vascular disease [11], contrast-induced kidney disease [12], and ischemia-reperfusion injury [13]. TMZ was first discovered to improving energy metabolism by inhibiting long-chain 3-ketoacyl-CoA thiolytic enzymes in the mitochondria, thereby limiting the aerobic oxidation of fatty acids [14–16]. Numerous subsequent studies have revealed that TMZ also plays various other essential roles, such as inhibiting cardiomyocyte apoptosis, reducing the production of reactive oxygen species, and improving myocardial interstitial fibrosis and anti-inflammation [17–22]. The latest clinical research has demonstrated that TMZ therapy during the perioperative period relieves endothelial dysfunction in patients with unstable angina induced by percutaneous coronary intervention [23]. However, the underlying mechanism by which TMZ regulates myocardial angiogenesis has not yet been revealed. Therefore, Our work studied the regulatory effects of TMZ on angiogenesis and investigated the activation of Akt, nuclear translocation of HSF1 and sequential VEGF-A accumulation in in endothelial cells, aiming to clarify the underlying mechanism of TMZ in alleviating myocardial angiogenesis dysfunction and heart failure.

## Materials and methods

### Animal model and ethics approval

Male C57 mice (purchased from Beijing Charles River Laboratories) were used in this study. All animal experiments were conducted using procedures approved by the Institutional Animal Care in accordance with the NIH Guide for the Care and Use of Laboratory Animals. The protocol was approved by the Committee on the Ethics of Animal Experiments of the Animal Research Committee of Tongji College. Twenty-four mice included in the experiment were divided into the following four groups: sham, transverse aortic constriction (TAC), TAC + trimetazidine (TMZ (HY-B0968A, MedChemExpress, NJ, USA)), and TAC + TMZ + KRIBB11 (HY-100872, MedChemExpress, NJ, USA) groups, with six mice in each group. After surgery, mice were administered either TMZ at 100 µL/2.8 mg or 100 µL PBS by gavage. No mice died after these treatment. Four weeks after surgery, ultrasound and histological staining were used to detect changes in the heart.

### Transverse aortic constriction (TAC)

TAC surgery was performed on 12-week-old male C57BL/6J mice as described previously [24]. Briefly, the mice were anesthetized and placed in a supine position on an operating table. After tracheal intubation, the mice were artificially ventilated at a tidal volume of 2–3 mL/h, and a respiratory rate of 90–110 beats per minute (BPM) was maintained using a dedicated mouse ventilator. An incision was made in the chest wall at the second intercostal space. The mouse aorta was then hooked out with a homemade hook connected with a string through the incision, and the tissues surrounding the blood vessel were separated. Next, a 27G needle was placed between the aorta and ligature. After ligation, the needle was withdrawn, and the chest cavity was closed and sutured layer-by-layer. Cardiac ultrasound was performed 2 weeks after TAC to validate the success of the model.

### Ultrasound detection

Mice were anesthetized with 2% isoflurane and their limbs were firmly fixed on the console with paper tape, and then the flow rate of 2% isoflurane was adjusted, while screening HR, to control the HR between 550 and 600 BPM. The Visualsonics Vevo 1100 system (Vevo 1100, FUJIFILM VisualSonics, Japan) and a 30 Hz probe were used to detect the left ventricular long axis and short axis sections of the mice and collect relevant information including left ventricular internal diameter, left ventricular anterior wall thickness, left ventricular posterior wall thickness, and ejection fraction from at least three cardiac cycles.

### Cell culture

HUVECs (Our laboratory preserved cell lines) were seeded onto 6-well plates containing RPMI-1640 medium (11875101, Thermofisher Scientific, MA, USA) supplemented with 10% FBS (10091, Thermofisher Scientific, MA, USA) and then placed in a 37 °C cell incubator. The medium was replaced with RPMI-1640 medium without FBS 24 h before intervention. After incubation with epinephrine (PE, 100 µM, HY-B0447B, MedChemExpress, NJ, USA), TMZ (5 µM), KRIBB11 (10 µM), and AZD5363 (10 nM, HY-15431, MedChemExpress, NJ, USA) for 24 h, the cells were collected for subsequent experiments.

### Molecular docking of Akt and TMZ

The structure of Akt (PDB:6S9W) (resolution: 2.30 Å) was downloaded from the protein data bank (http://www.rcsb.org/), and the structure of TMZ was constructed using ChemSketch 12.0 (ACD Systems, Canada). These two structures were imported into the program Schrodinger Suites 2018 (Schrodinger, Inc. CA, USA). The Protein Preparation Wizard tool [25] was used to optimize the structure of Akt by removing water molecules and arranging bond orders. Likewise, the structure of TMZ was optimized using the Ligand Preparation tool to neutralize the structure and generate a possible tautomer. Additionally, conformation alteration was allowed in the docking calculation. Next, a ligand docking tool was used to calculate the docking score and determine the possible bonding site and interactions between the ligand and protein.

### Immunofluorescence staining

The distribution of HSF1 was assessed by immunofluorescence as described previously [26]. Briefly, the cells were fixed with 4% paraformaldehyde (G1101-500ML, Servicebio, Wuhan, China) for 15 min, permeabilized with 1% Triton X-100 (G5060-100ML, Servicebio, Wuhan, China) for 10 min, and incubated overnight at 4 °C with anti-HSF1 primary antibody (A13765, 1:100, Abclonal, Wuhan, China). Next, the cells were incubated with Cy3-labeled goat anti-rabbit antibody (BA1032, Boster, Wuhan, China) for 1 h and DAPI (AR1177, Boster, Wuhan, China) for 15 min at room temperature. The distribution of HSF1 was then observed by fluorescence microscopy (IX73-A12FL/PH, OLYMPUS, Japan).

### CCK8 assay

HUVEC proliferation was detected using CCK8 assay as described previously [27]. Briefly, the cells to be tested were scraped from a 6-well plate to prepare a cell suspension and seeded onto a 96-well plate at a density of 1000 cells per well. The 96-well plate was incubated at 37 °C for 4–6 h, and after 1–4 h of incubation with 10 µL of CCK8 (CCK8, Dojindo Laboratories, Japan), the absorbance at 450 nm was measured.

### 5-ethynyl-2′-deoxyuridine (EdU) staining

The proliferation of HUVECs was detected with EdU staining as described previously [28]. Briefly, 2 mL of medium containing 10 µM EdU (ab222421,abcam,UK) was added to each well of a 6-well plate. After incubating the cells for 2 h at 37 °C, the culture medium was discarded, 4% paraformaldehyde fixative and 0.5% Triton X-100 for permeation were added sequentially, and the cells were incubated in Apollo staining solution at 37 °C for 30 min in the dark. Next, the cells were observed using a fluorescence microscope.

### Dual-luciferase reporter assay

The promoter regions of wild-type and mutant *VEGF-A* genes were cloned into the pGL4 basic luciferase reporter vector, and *HSF1* was cloned into the pcDNA3.1 (+) plasmid (V79020, Thermofisher Scientific, MA, USA). All vectors were verified by sequencing. Mouse endothelial cells were seeded onto a 24-well culture dish, and each well was transfected with wild-type or mutant *VEGF-A* promoter luciferase reporter gene (0.5 µg) and HSF1 (0.5 µg). Forty-eight hours after transfection, cell lysates were collected, and the Dual-Luciferase Reporter Assay kit (E2920, Promega, USA) was used to analyze the firefly and *Renilla* luciferase activity. The experiment was repeated three times. The ratio of firefly-to-*Renilla* luciferase activity was used as a standardization index for the luciferase activity of each group.

### Electrophoretic mobility shift assay (EMSA)

The double-stranded VEGF-A DNA probe with or without the 5′-biotin label was used in EMSA. Equimolar complementary strands were mixed and gradually cooled to room temperature after incubating for 5 min at 95 °C. According to the manufacturer’s instructions (GS009, Beyotime, Shanghai, China), the double-stranded DNA probe and nuclear extracts were thoroughly mixed in a 10-µL reaction system. The complex was then separated on a 6% polyacrylamide gel containing 0.5× TBE and transferred to a positively charged nylon membrane. After UV crosslinking, the labeled DNA probes were detected with HRP-streptavidin.

### Chromatin immunoprecipitation (CHIP)-qPCR

ChIP analysis was performed using the ChIP kit (P2078, Beyotime, Shanghai, China) according to the manufacturer’s instructions as described previously [29]. Cells overexpressing HSF1 or the control vector were crosslinked with 1% formaldehyde at 37 °C for 15 min. The resulting cell pellet was then collected. The nucleus extracts were obtained with the nuclear protein and cytoplasmic protein preparation kit (P1200, Applygen, Beijing, China), and the chromatin was cut into a length of ~200–400 bp with ultrasound. The sheared nuclear extracts were incubated with anti-HSF1 antibody (sc-17757X, Santa Cruz, CA, USA) and protein A/G agarose beads (20422, Thermofisher Scientific, MA, USA) overnight at 4 °C. Next, the protein A/G agarose beads–antibody–protein complex was collected and separated with elution buffer after de-crosslinking. DNA was extracted with a DNA purification kit (DP304, TIANGEN, Beijing, China), and the precipitated DNA fragments were analyzed by real-time quantitative PCR. Primers of VEGF-A (sense: 5′-GAAGATGTGGAGAGTTGGAGGAA-3′; anti-sense: 5′-CCTGCGTGATGATTCAAACC-3′). The nuclear extracts were used as a positive control, and the nuclear extracts incubated with anti-IgG antibody (ab205718, abcam, UK) were used as a negative control. Each assay was repeated for three times.

### Scratch wound healing assay

Scratch wound healing assay was performed as described previously [30]. 1 × 10^6^ cells/well were seeded into a 6-well plate that has been coated with extracellular matrix (ECM) substrates (50 μg/mL poly-*L*-lysine, C0313-5mg, Beyotime, Shanghai, China). The monolayer was scratched in a straight line by a pipette tip. Detached cells were then removed. Then remaining cells were cultured in a fresh medium supplemented with PE, TMZ, and HSF1 inhibitor for 12 h. Mitomycin C (10 μg/mL, HY-13316, MedChemExpress, NJ, USA), was also added to inhibit DNA replication. After the incubation, cell monolayers were fixed with 3.7% paraformaldehyde for 15 min and photographed at 0 h, 12 h post-wounding. Wound area was then calculated by manually tracing the cell-free area in images using ImageJ software and by the following formula: wound closure % = (*A*_t = 0 h_ − *A*_t = 12 h_)/*A*_t = 0 h_ × 100, where *A*_t = 0 h_ is the initial area (time zero), and *A*_t =12 h_ is the area of the wound measured 12 h post scratch.

### Tube formation assay

Tubule formation assay was conducted as previously described [31]. Briefly, before seeding 2 × 10^4^ cells/well, the pipet and 96-well plates were precooled and coated with 50 μL of Matrigel (356234, BD Biosciences, Bedford, MA, USA) for 30 min at 37 °C. After incubation for 6 h in 100 μL endothelial culture medium containing different chemicals (PE 100 µM, AZD5363 10 nM, KRIBB11 10 µM) in incubator, HUVECs were captured by a bright filed microscope. Total tube length was analyzed to quantify the tube formation ability.

### Western blot

HUVECs were lysed on ice for 30 min with the cell lysate. The protein concentration was measured using the BCA method (P0011, Beyotime, Shanghai, China) before being subjected to electrophoresis and transferred to a PVDF membrane (IPVH00010, Millipore, Germany). The PVDF membrane was incubated with antibodies against p-HSF1, HSF1, VEGF, p-Akt, Akt, and GAPDH overnight at 4 °C and finally exposed and photographed. The antibody information was listed in Supplementary Table S1.

### Co-immunoprecipitation (Co-IP)

Co-IP experiments were performed as described previously [32]. Cells that had been transfected with the indicated plasmids were lysed with IP lysis buffer (P0013J, Beyotime, Shanghai, China). Before performing SDS-PAGE, the pre-cleared cell lysate was incubated with an appropriate amount of primary antibody at 4 °C overnight. Protein A/G agarose beads (sc-2003, Santa Cruz, CA, USA) were then used to precipitate the target protein. After performing SDS-PAGE, the proteins were immediately transferred to the PVDF membrane and incubated with antibodies against HSF1, Akt, and p-Akt. The protein of interest was then visualized by chemiluminescence using the Tanon system (Tanon 5200, Shanghai, China). The antibody information was listed in Supplementary Table S1.

### Statistical analysis

All data are expressed as the mean ± SEM. The statistic’s normality was tested by D’Agostino and Pearson omnibus normality test. Comparison between two groups was analyzed by Student’s *t* test. Multiple group comparisons were analyzed by one-way or two-way ANOVA, followed by Tukey’s multiple comparison test using GraphPad Prism 8.0 software. Mann–Whitney test and Kruskal–Wallis test would be applied when the data did not show a normal distribution. *P* < 0.05, represents statistically significant values.

## Results

### TMZ alleviated angiogenesis dysfunction and improved heart function in pressure overload-induced cardiac hypertrophy mice

Mice (12 weeks old) were administered TMZ by gavage for 28 consecutive days following TAC surgery to investigate the role of TMZ in promoting angiogenesis. The ultrasound (Supplementary Fig. S1a–c), cardiac morphology (Supplementary Fig. S1a), and heart weight/tibia length (HW/TL) (Supplementary Fig. S1d) results demonstrated that TMZ effectively relieved myocardial hypertrophy under pressure overload, which manifested with increased heart weight, enlarged heart chambers, and increased thickness of the anterior and posterior walls of the left ventricular myocardium. The levels of cardiac hypertrophy markers, atrial natriuretic peptide, and B-type natriuretic peptide, also decreased with TMZ treatment (Supplementary Fig. S1f–h).

**Fig. 1:**
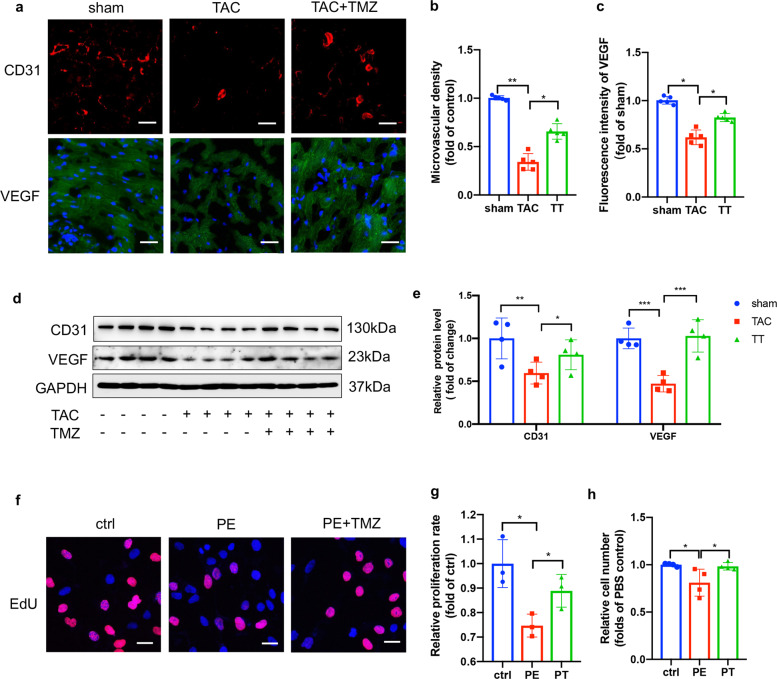
TMZ alleviated angiogenesis dysfunction in pressure overload-induced cardiac hypertrophy mice. **a** Immunohistochemical staining of CD31 and VEGF was presented from left ventricular myocardium. Scale bar = 50 μm. **b**, **c** Quantification analysis of (**a**), *n* = 5. **d** Western blotting of CD31 and VEGF, GAPDH was used as a loading control, *n* = 3. **e** Quantification analysis of (**d**). **f** Representative EdU staining images of HUVECs. Scale bar = 20 μm. **g** Quantification analysis of (**f**). **h** CCK8 assay of HUVECs in indicated groups. Sham sham operation group, TAC TAC group, TT TAC + TMZ group, mice subjected to TAC surgery and treated with TMZ, TTHi TAC + TMZ + HSFi group, TAC mice were treated with HSF1 inhibitor (KRIBB11) and TMZ, Ctrl, PBS group, PE Norepinephrine group, PT PE + TMZ group, cells treated with PE and then treated with TMZ, PTHi PE + TMZ + HSFi group, cells were treated with HSF1 inhibitor (KRIBB11), TMZ and PE. Data were analyzed by two-way ANOVA analysis using GraphPad Prism 8 software (*P < 0.05, **P < 0.01; ***P < 0.01; ****P < 0.0001 vs. indicated group).

Immunofluorescence staining for CD31 was performed to detect the extent of blood vessel formation. As shown in Fig. 1a, the blood vessels decreased significantly in the TAC group compared with the sham group, whereas abundant blood vessels appeared in the TMZ treatment group. The quantification of angiogenesis, analyzed by the number of cells positively stained for CD31, verified the angiogenesis-promoting effects of TMZ (Fig. 1b). Western blotting also showed increased expression of CD31 and VEGF in mouse hearts treated with TMZ (Fig. 1d, e). The angiogenesis-stimulating function of TMZ in vitro was confirmed using proliferation assays. As expected, fewer EdU^+^ cells were observed in the PE group. However, TMZ accelerated cell proliferation, as shown by the much higher number of EdU^+^ cells (Fig. 1f, g). Our data suggest that TMZ augmented the angiogenic responses and improved the heart function of pressure overload-induced cardiac hypertrophy mice.

### Inhibition of HSF1 activation restrained the angiogenic effect of TMZ

**Fig. 2:**
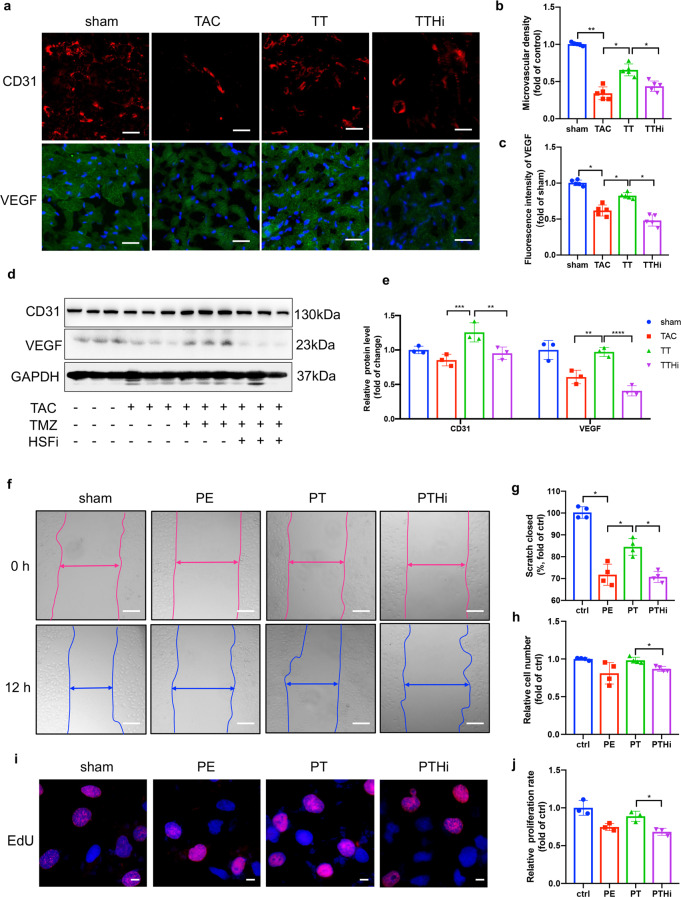
HSF1 played a critical role in TMZ-mediated cardiac angiogenesis improvement. **a** Immunohistochemical staining of CD31 and VEGF was presented from left ventricular myocardium. Scale bar = 50 μm. **b**, **c** Quantification analysis of (**a**), *n* = 5. **d** Western blotting of CD31 and VEGF, GAPDH was used as a loading control, *n* = 3. **e** Quantification analysis of (**d**). **f** Representative migration images of HUVECs in 12 h. Scale bar = 200 μm. **g** Relative scratch closed area of (**f**). **h** CCK8 assay of HUVECs in indicated groups. **i** Representative EdU staining images of HUVECs. Scale bar = 20 μm. **j** Quantification analysis of (**i**). Sham sham operation group, TAC TAC group, TT TAC + TMZ group, mice subjected to TAC surgery and treated with TMZ, TTHi TAC + TMZ + HSFi group, TAC mice were treated with HSF1 inhibitor (KRIBB11) and TMZ, Ctrl PBS group, PE Norepinephrine group, PT PE + TMZ group, cells treated with PE and then treated with TMZ, PTHi PE + TMZ + HSFi group, cells were treated with HSF1 inhibitor (KRIBB11), TMZ and PE, Data were analyzed by two-way ANOVA analysis using GraphPad Prism 8 software (*P < 0.05, **P < 0.01; ***P < 0.01; ****P < 0.0001 vs. indicated group).

In our previous study, HSF1 was demonstrated to be critically involved in cardiac angiogenesis [33, 34]. We then asked whether HSF1 was responsible for the angiogenesis-stimulating effect of TMZ. As detected by fluorescence staining and Western blot, fewer blood vessels and lower VEGF and CD31 expression were observed in the presence of HSF1 inhibitors (Fig. 2a–e). Correspondingly, to assess the effects of HSF1 on endothelial cells, a scratch wound assay was applied to measure cell migration ability. The results show that HSF1 inhibitor treatment markedly weakened the motility of HUEVCs, as determined by the larger wound closure area (Fig. 2f, g). The proliferation of HUEVCs was quantified by CCK8 analysis, EdU staining, and tube formation assay (Supplementary Fig. S3a, b). The results revealed that TMZ treatment caused a marked increase in proliferation. However, the effect was suppressed by the HSF1 inhibitor (Fig. 2h–j, Supplementary Fig. S2a, b). Collectively, these data suggest that TMZ enhances cardiac angiogenesis by HSF1.

### TMZ promoted the binding of HSF1 with the VEGF-A promoter region

**Fig. 3:**
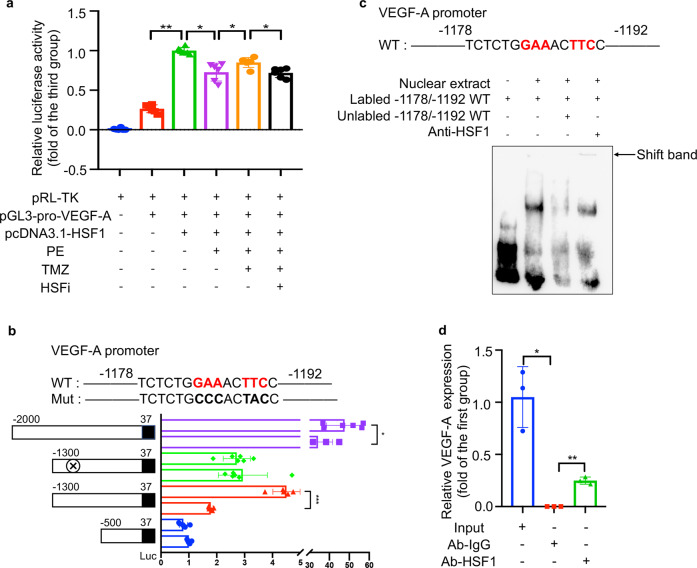
HSF1 bound with VEGF-A promoter region. **a** Luciferase activity of pGL3-f reporter plasmids in indicated groups. **b** Luciferase activity of pGL3 (−2000/+37), pGL3 (−1300/+37), mutation pGL3 (−1300/+37), and pGL3 (−500/+37) reporter plasmids stimulated by pcDNA3.1-HSF1, mutation region located in −1192 to −1178. **c** Representative EMSA images with biotin-labeled DNA oligonucleotides VEGF-A-1192/−1178-WT. **d** Verification of the interaction of HSF1 and the promoter region of VEGF-A by ChIP-qPCR. Ctrl PBS group, PE Norepinephrine group, PT PE + TMZ group, cells treated with PE and then treated with TMZ, PTHi PE + TMZ + HSFi group, cells were treated with HSF1 inhibitor (KRIBB11), TMZ and PE. Data were analyzed by two-way ANOVA analysis using GraphPad Prism 8 software (*P < 0.5; **P < 0.01, vs. indicated group).

To further investigate the relationship between HSF1 and angiogenesis, we predicted several possible binding molecules for HSF1 from the JASPAR website, and found a specific binding site for HSF1 in the promoter region of VEGF-A. Consequently, we determined whether HSF1 bound to the VEGF-A promoter region, and if TMZ could enhance this binding. Dual-luciferase reporter assay was then carried out. The results revealed that HSF1 did combine with the promoter region of VEGF-A. TMZ strengthened this interaction, whereas the effect was reduced after HSF1 activity was inhibited (Fig. 3a). These data suggest that TMZ promoted the binding of HSF1 with the VEGF-A promoter region.

Four fragments of the VEGF-A promoter region (−2000/+37, −1300/+37 mutant, −1300/+37 WT, and −500/+37) were then cloned into a pGL3 fluorescent reporter gene vector to validate the HSF1–VEGF-A binding site. HSF1 significantly increased the fluorescence intensity of the –2000/+37 bp and –1300/+37 bp regions, while the other two regions showed no changes, indicating that the HSF1–VEGF-A promoter region binding site is located in the 1300 bp/500 bp region; more accurately, the position is −1192 bp/−1178 bp (Fig. 3b). The regulatory effects of the −1192 bp/−1178 bp region were further evidenced by EMSA assay and CHIP-qPCR assay (Fig. 3c, d).

### TMZ activated HSF1 and promoted its nuclear translocation via Akt

**Fig. 4:**
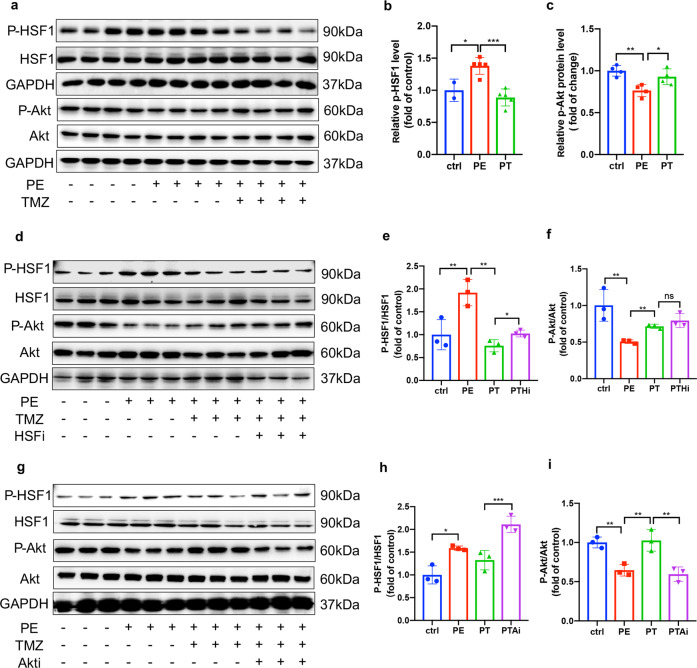
TMZ triggered HSF1 via the activation of Akt. **a** Western blotting images of the phosphorylation of Akt and HSF1 in HUVECs exposed to PE with or without TMZ, GAPDH was used as a loading control. **b** Quantitative analysis of Western blotting images of p-HSF1 and HSF1 in (**a**). **c** Quantitative analysis of Western blotting images of p-Akt and Akt in (**a**). **d** Western blotting analysis of the phosphorylation of Akt and HSF1 in HUVECs exposed to PE and TMZ with or without HSF1 inhibitor (KRIBB11), GAPDH was used as a loading control. **e** Quantitative analysis of Western blotting images of p-HSF1 and HSF1 in (**d**). **f** Quantitative analysis of Western blotting images of p-Akt and Akt in (**d**). **g** Western blotting analysis of the phosphorylation of Akt and HSF1 in HUVECs exposed to PE and TMZ with or without Akt inhibitor (AZD5363), GAPDH was used as a loading control. **h** Quantitative analysis of Western blotting images of p-HSF1 and HSF1 in (**g**). **i** Quantitative analysis of Western blotting images of p-Akt and Akt in (**g**). Ctrl PBS group, PE Norepinephrine group, PT PE + TMZ group, cells treated with PE and then treated with TMZ, PTHi PE + TMZ + HSFi group, cells were treated with HSF1 inhibitor (KRIBB11), TMZ and PE, PTAi: PE + TMZ + Akti group, cells were treated with Akt inhibitor (AZD5363), TMZ and PE. Data were analyzed by two-way ANOVA analysis using GraphPad Prism 8 software (*P < 0.5; **P < 0.01; ***P < 0.01 vs. indicated group).

We previously identified that TMZ activated Akt in a dose-dependent manner from 1 to 5 µM (Supplementary Fig. S2). To investigate whether HSF1 and Akt respond to TMZ treatment under pressure overload, PE-treated HUVECs were extracted, and Western blot was then used to detect changes in the phosphorylation level of HSF1 and Akt. As expected, HSF1S303 expression was elevated under PE treatment and reduced upon TMZ stimulation. In contrast, AktT473 expression decreased in response to PE treatment, while it increased in the presence of TMZ. These results indicate that HSF1 and Akt synchronously respond to TMZ (Fig. 4a–c).

Specific inhibitors of Akt (AZD5363) and HSF1 (KRIBB11) were then used in subsequent experiments to further clarify the specific regulatory relationship between HSF1 and Akt. As shown in Fig. 4d–i, TMZ could not activate HSF1 and promote its nuclear translocation following Akt inhibition, whereas it could still activate Akt when HSF1 was inhibited. These opposite results indicate that Akt is upstream of HSF1.

**Fig. 5:**
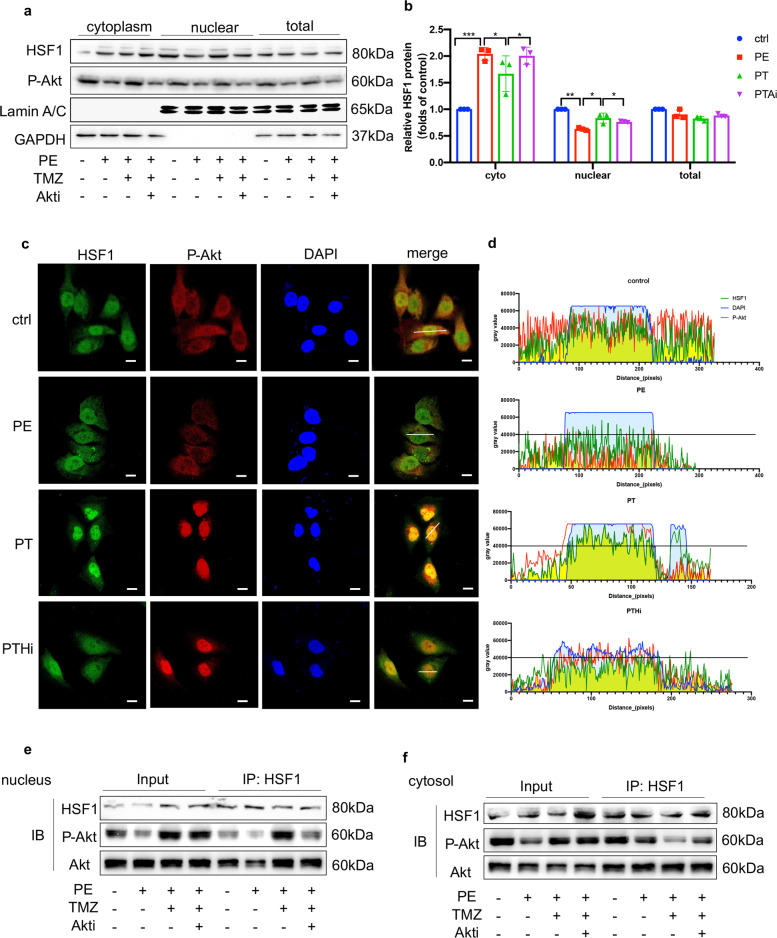
TMZ promoted the nuclear translocation and binding of Akt and HSF1. **a** Western blotting analysis of the phosphorylation of Akt and HSF1 in the cytoplasm and nuclear of HUVECs exposed to PE and TMZ with or without Akt inhibitor (AZD5363), GAPDH was used as a total and cytoplasm loading control, Lamin A/C was used as a nuclear loading control. **b** Quantitative analysis of Western blotting images in (**a**). **c** Immunofluorescence staining with the p-Akt antibody (red) and HSF1 (green) of HUVECs. **d** Quantitative analysis of immunofluorescence intensity in indicated line. **e**, **f** Immunoprecipitation assays showing the interactions between p-Akt1 and HSF1 in HUVECs. Nuclear extracts and cytosol of HUVECs were obtained from indicated groups. Ctrl PBS group, PE Norepinephrine group, PT PE + TMZ group, cells treated with PE and then treated with TMZ, PTHi PE + TMZ + HSFi group, cells were treated with HSF1 inhibitor (KRIBB11), TMZ and PE, PTAi PE + TMZ + Akti group, cells were treated with Akt inhibitor (AZD5363), TMZ and PE. Data were analyzed by two-way ANOVA analysis using GraphPad Prism 8 software (*P < 0.5; **P < 0.01; ***P < 0.01 vs. indicated group).

Next, HUVEC nucleoproteins and cytoplasmic proteins were separated to detect changes in the nuclear translocation of HSF1. Under TMZ treatment, the nuclear signals of HSF1 and p-Akt were significantly enhanced. However, they faded with the addition of the Akt inhibitor (Fig. 5a, b). Immunofluorescence analysis of HSF1 and p-Akt also showed that PE inhibited the phosphorylation of Akt, as well as the nuclear translocation of HSF1, while TMZ reversed these effects. The overlapping yellow parts in the nucleus suggest that HSF1 and Akt have a mutual binding relationship (Fig. 5c, d). Their binding relationship was also verified by Co-IP assay. As shown in Fig. 5e, f, HSF1 and Akt bound to each other without stimulation. The binding of Akt/p-Akt and HSF1 in the nucleus was significantly enhanced after TMZ treatment, and remarkably reduced when Akt was inhibited. These results reveal that Akt and HSF1 have a direct binding relationship, and that TMZ promotes the synergistic nuclear translocation of Akt and HSF1 by activating Akt.

### Trimetazidine directly bound to Akt and activated Akt

**Fig. 6:**
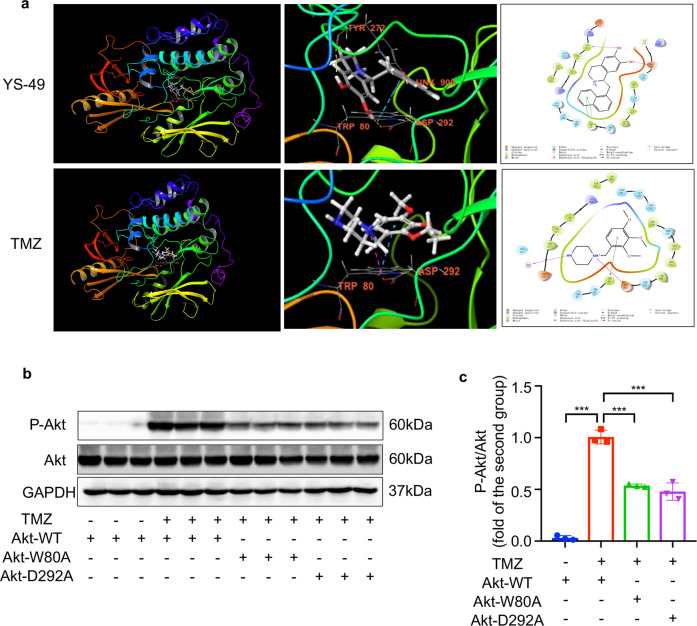
TMZ bound to Akt in Trp80 and Asp292. **a** Upper, known agonist of Akt (YS-49) binds to Akt in Trp80, Asp292, and Tyr272. Lower, TMZ binds to Akt in Trp80 and Asp292. **b** Western blotting analysis of the phosphorylation of Akt in 293T cells transfected with indicated plasmids. GAPDH was used as a loading control. **c** Quantitative analysis of Western blotting images of (**b**). Data were analyzed by Student’s *t* test using GraphPad Prism 8 software (***P < 0.001 vs. indicated group).

As Akt was determined to be upstream of HSF1, we then asked whether TMZ directly binds to Akt. Schrodinger Suites 2018 was applied to examine the molecular docking of TMZ and Akt. YS-49, a known Akt agonist, was used as a positive control; as shown in Fig. 6a, YS-49 was located inside and completely wrapped by Akt. It combined with the Asp292, Trp80, and Tyr272 sites in Akt, forming two hydrogen bonds with Asp292 and Tyr272, and a pi–pi bond with Trp80, yielding a binding affinity of −9.04 kcal/mol. Similar to YS-49, TMZ was also located inside Akt and wrapped by it. Among the three core sites to which YS-49 and Akt bound, two residues bound with TMZ; TMZ formed a hydrogen bond with Asp292, and a pi–pi bond with Trp80, yielding a binding affinity of −5.995 kcal/mol. TMZ and YS-49 overlapped on two binding sites with relatively high binding affinities, illustrating that TMZ directly activates Akt. To validate whether Trp80 and Asp292 are critical amino acids, two mutational plasmids (Akt^W80A^ and Akt^D292A^) were constructed. Fig. 6b, c shows that neither Akt^W80A^ nor AktD^292A^ responded to TMZ, unlike Akt^WT^, suggesting that these two amino acids played a vital role in the activation of Akt by TMZ. Taken together, the above results show that TMZ directly activates Akt in endothelial cells via its binding with Trp80 and Asp292.

## Discussion

**Fig. 7:**
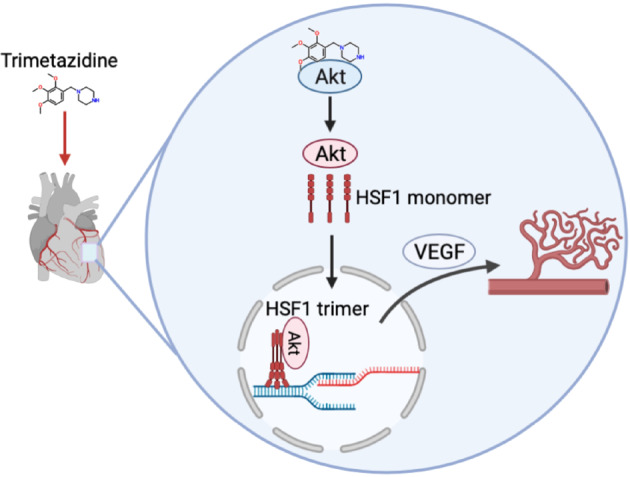
Schematic overview of the proposed mechanism. TMZ activated Akt, and promoted the binding of Akt to HSF1, leading to the activation and nuclear translocation of HSF1. Activated HSF1 bound with the promoter region of VEGF-A, and eventually promoted its expression and angiogenesis.

In this study, we demonstrate that TMZ binds to the Trp80 and Asp292 sites of Akt and subsequently activates Akt. It promotes the binding of Akt and HSF1, as well as the nuclear translocation of HSF1, by activating Akt. TMZ enhances HSF1–VEGF-A promoter region binding, thereby improving the angiogenesis dysfunction caused by pressure overload and promoting cardiac function. Our proposed model for the mechanism by which TMZ exerts its protective effect against cardiac angiogenesis is summarized in Fig. 7.

TMZ is widely used in the clinical treatment of cardiovascular diseases, including angina pectoris, ischemia-reperfusion, diabetic cardiomyopathy, and heart failure [35, 36]. TMZ is reported to exert its protective effects through various mechanisms. It prevents diabetic cardiomyopathy by inhibiting NOX2/TrPC3-induced oxidative stress [37], and protects against atherosclerosis by reducing reactive oxygen species production and regulating smooth muscle proliferation [38]. In addition, TMZ has also been implicated in the regulation of mitochondrial metabolism, mitochondrial biosynthesis, and mitochondrial fission/fusion [39, 40]. In contrast to previous studies, our study confirms that TMZ attenuates cardiac hypertrophy and heart failure by improving angiogenesis dysfunction. TMZ treatment increases blood vessel density and enhances VEGF expression, both in vivo and in vitro.

Akt is a serine/threonine kinase member of the AGC protein family and is widely involved in many cellular processes, including energy metabolism, angiogenesis, cell proliferation, and tumor growth. Thus, Akt is considered one of the most important signaling molecules in eukaryotic cells [41]. Trp80 and Asp292 are the two key amino acid residues that regulate Akt activity. Trp80 is the critical residue in the docking of MK-2206 with Akt [42]. When Trp80 is mutated to form alanine, Akt is resistant to its inhibitors [43]. Monocrotophos organophosphate pesticides inhibit Akt owing to the strong hydrogen bond between the Glu234 site of the monocrotophos and the Asp292 site of Akt [44]. As a well-known metabolism modulating agent, TMZ also acts on Akt. For example, TMZ upregulates p-Akt in myocardial ischemia/reperfusion injury [13], and protects umbilical cord mesenchymal stem cells against hypoxia and serum deprivation-induced apoptosis by activating Akt [45]. Our previous study also revealed that TMZ activates Akt in a dose-dependent manner. However, none of these studies clarified the direct interaction between Akt and TMZ. Using the molecular docking software Schrodinger Suites 2018, we found that, similar to the working mode of YS-49 (an Akt agonist [46]), TMZ formed a hydrogen bond and a pi–pi bond with the Asp292 and Trp80 sites of Akt, respectively. Further experiments conducted in HUVECs provided solid evidence that both Asp292 and Trp80 are crucial for the activation of Akt.

As a typical transcription factor, the activity of HSF1 was determined by its abundance, protein modification, and subcellular distribution [47]. There were at least 12 phosphorylation sites in HSF1, of which Ser307 and Ser303 are well-established inhibitory phosphorylation sites. This implies that HSF1 activity decreases when phosphorylation levels of Ser307 and Ser303 increase [48]. Inactive HSF1 localizes in the cytosol and nucleus as a monomer. When HSF1 is activated, it changes its form from a monomer to a trimer and then translocates from the cytosol into the nucleus [47]. In the present study, the inhibitory phosphorylation level of HSF1 in HUVECs was significantly downregulated by TMZ treatment, accompanied by its enhanced nuclear accumulation, indicating that HSF1 was activated by TMZ. However, the activation of HSF1 was counterbalanced by the addition of Akt inhibitor, suggesting that HSF1 is not directly activated by TMZ, but is rather activated via Akt.

Several molecules are involved in the regulation of HSF1 activity. ERK inhibits HSF1 by directly phosphorylating HSF1 at Ser307 [49], while GSK3β exerts its inhibitory effect on HSF1 by phosphorylating Ser303 [50]. Sirt1 acts as an upstream molecule of HSF1 to reduce the acetylation level of HSF1 and activate it [51]. In the present study, enhanced HSF1 activity was accompanied by increased Akt activity. Inhibiting Akt restrained the activation of HSF1, whereas inhibiting HSF1 had no effects on the activity of Akt. These results suggest that Akt is critically involved in the regulation of HSF1 activity. Co-IP and immunofluorescence experiments further confirmed that Akt binds with HSF1. When Akt was activated by TMZ, it cooperated with HSF1 to aid its translocation to the nucleus, further exerting its regulatory effects.

HSF1 plays an essential role in autophagy, aging, development, metabolism, and immunity [47, 52]. In the heart, HSF1 induces the apoptosis of senescent myocardial mitochondria by activating Omi/HtrA2 transcription [53], and acts as the main defender against palmitic acid-induced myocardial ferroptosis [54]. The latest research has confirmed that HSF1 promotes myocardial angiogenesis through the HIF-1α/VEGF pathway [33, 55]. Consistently, in our study, HSF1 activity increased with TMZ treatment under pressure overload, accompanied by an increase in myocardial blood vessel density, whereas the blood vessel density decreased with HSF1 inhibition.

Myocardial angiogenesis is regulated by a series of growth-promoting factors, including VEGF, angiopoietin-1 and -2, fibroblast growth factors, transforming growth factors, and platelet-derived growth factors [7]. Among them, VEGF is the primary regulator of myocardial angiogenesis [56]. VEGF-deficient mice were more likely to transition from pathological myocardial hypertrophy to heart failure after TAC surgery [57]. In contrast, VEGF overexpression improved the contractility of failing hearts [58]. The present study reveals that, as a transcription factor, HSF1 binds with heat shock elements in the promoter region of VEGF-A and participates in the regulation of VEGF expression, providing direct evidence of the close relationship between HSF1 and angiogenesis.

Owing to technical limitations, this study has several shortcomings. First, instead of generating HSF1-KO mice, we intraperitoneally injected the mice with the HSF1 inhibitor, KRIBB11. KRIBB11 may have potential off-target effects. Second, we demonstrated that TMZ formed hydrogen and pi–pi bonds with Akt using molecular docking analysis. However, we did not simulate the dynamic molecular conformation of Akt when combined with TMZ. Future studies are needed to elucidate the molecular conformational changes that occur when TMZ binds with Akt.

Our proposed model, based on the above data, is shown in Fig. 7. After TMZ enters the endothelial cells, it activates Akt by binding with its Asp292 and Trp80 residues. Then, the activated Akt activates and promotes the nuclear translocation of HSF1. By combining with the promoter region of VEGF-A, HSF1 stimulates the expression of VEGF-A, and ultimately attenuates angiogenesis dysfunction and promotes cardiac function under pressure overload. In summary, our study demonstrates that HSF1 mediates the protective effects of TMZ in promoting angiogenesis, and provides a new theoretical basis for the cardio-protective functions of TMZ.

## Supplementary information

Supplementary Table S1

Figure S1

Figure S2

Figure S3
