# Supplementary figures and images for: Trimetazidine enhances myocardial angiogenesis in pressure overload-induced cardiac hypertrophy mice through directly activating Akt and promoting the binding of HSF1 to VEGF-A promoter
Source: Acta Pharmacol Sin. 2022 Feb 25;43(10):2550–61. doi: 10.1038/s41401-022-00877-8 (PMC9525722; doi:10.1038/s41401-022-00877-8)

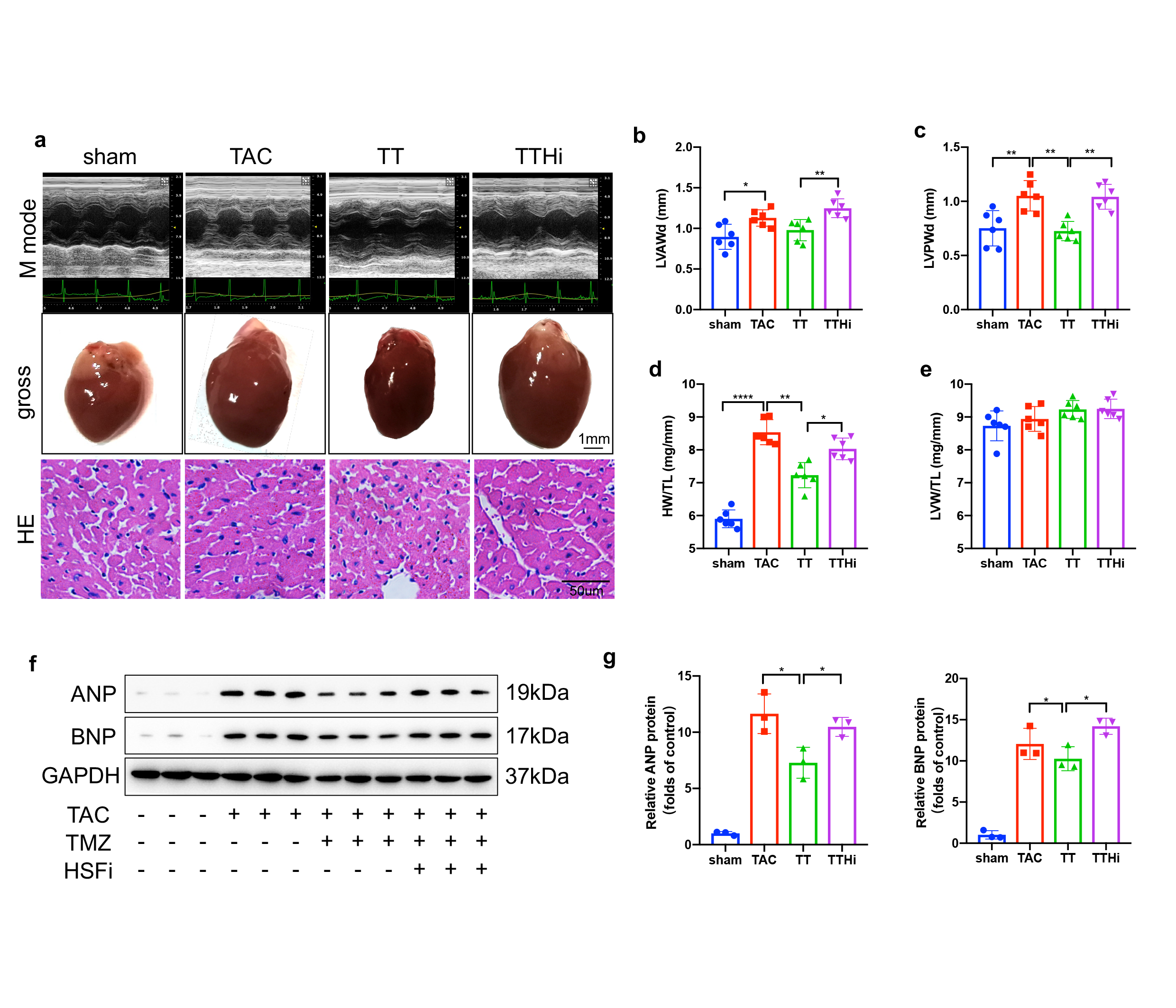

Supplement: Supplementary file 2 — Figure S1 [file 41401_2022_877_MOESM2_ESM.tiff]

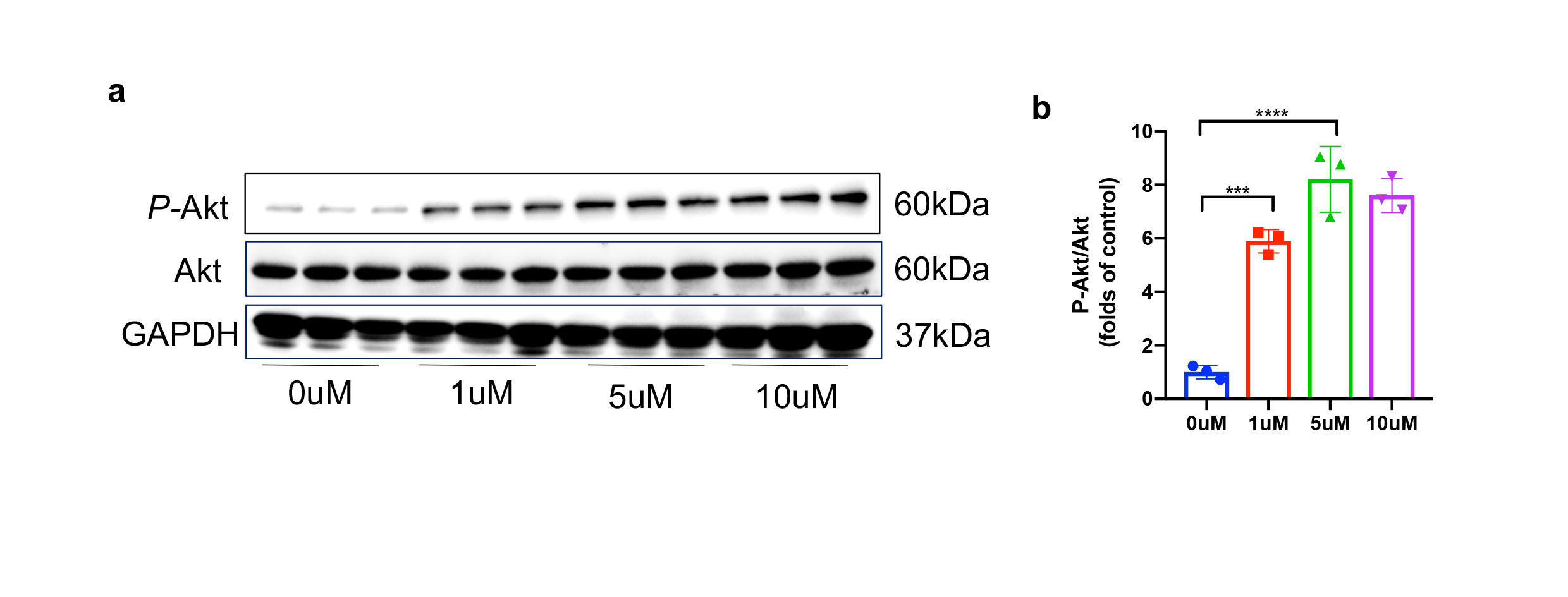

Supplement: Supplementary file 3 — Figure S2 [file 41401_2022_877_MOESM3_ESM.tiff]

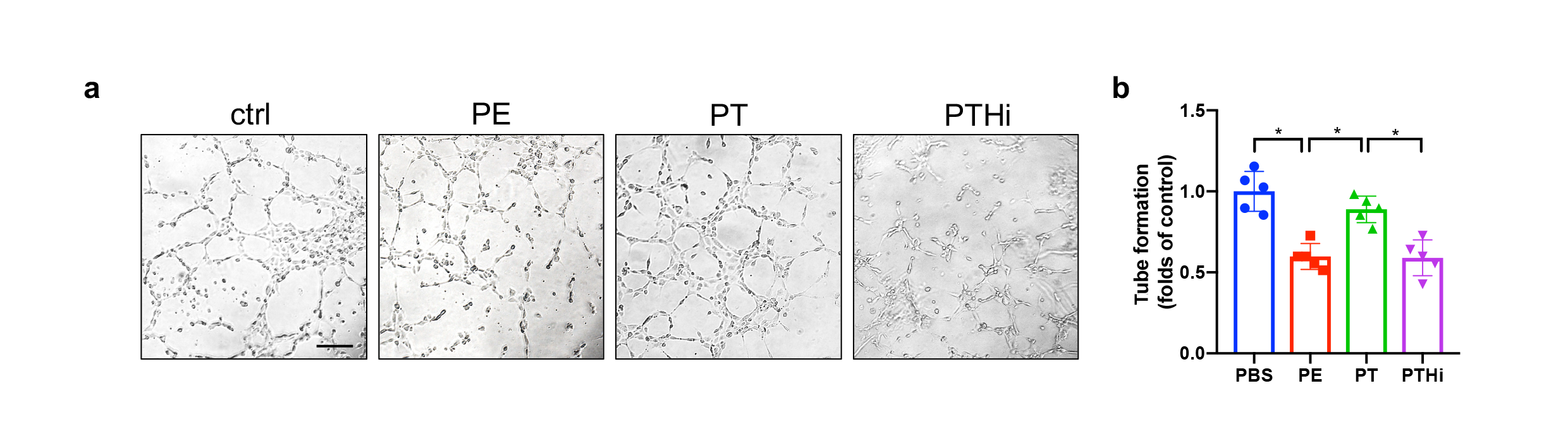

Supplement: Supplementary file 4 — Figure S3 [file 41401_2022_877_MOESM4_ESM.tiff]
